# Multilineage Potential Research of Bovine Amniotic Fluid Mesenchymal Stem Cells

**DOI:** 10.3390/ijms15033698

**Published:** 2014-02-28

**Authors:** Yuhua Gao, Zhiqiang Zhu, Yuhua Zhao, Jinlian Hua, Yuehui Ma, Weijun Guan

**Affiliations:** 1 Institute of Animal Sciences, Chinese Academy of Agricultural Sciences, Beijing 100193, China; E-Mails: gyhann@hotmail.com (Y.G.); yuehui_ma@hotmail.com (Y.M.); 2 College of Wildlife Resources, Northeast Forestry University, Harbin 150040, China; 3 Harbin Institute of Physical Education, Harbin 150008, Heilongjiang, China; E-Mails: chunyu_bai@hotmail.com (Z.Z.); baichunyu001@gmail.com (Y.Z.); 4 College of Veterinary Medicine, Shaanxi Centre of Stem Cells Engineering and Technology, Key Lab for Animal Biotechnology of Agriculture Ministry of China, Northwest A&F University, Yangling 712100, Shaanxi, China; E-Mail: jinlianhua@nwsuaf.edu.cn

**Keywords:** bovine, amniotic fluid mesenchymal stem cell, multiply differentiation

## Abstract

The use of amnion and amniotic fluid (AF) are abundant sources of mesenchymal stem cells (MSCs) that can be harvested at low cost and do not pose ethical conflicts. In human and veterinary research, stem cells derived from these tissues are promising candidates for disease treatment, specifically for their plasticity, their reduced immunogenicity, and high anti-inflammatory potential. This work aimed to obtain and characterize bovine amniotic fluid mesenchymal stem cells (AFMSC). The bovine AF from the amniotic cavity of pregnant gilts in the early stages of gestation (3- and 4-m-old bovine embryos) was collected. AFMSCs exhibit a fibroblastic-like morphology only starting from the fourth passage, being heterogeneous during the primary culture. Immunofluorescence results showed that AFMSCs were positive for β-integrin, CD44, CD73 and CD166, but negative for CD34, CD45. Meanwhile, AFMSCs expressed ES cell markers, such as Oct4, and when appropriately induced, are capable of differentiating into ectodermal and mesodermal lineages. This study reinforces the emerging importance of these cells as ideal tools in veterinary medicine; future studies aimed at a deeper evaluation of their immunological properties will allow a better understanding of their role in cellular therapy.

## 1. Introduction

Amniotic fluid was first isolated and studied during the beginning of the 20th century; these cells showed the ability to expand *in vitro* and to integrate into a scaffold [1,2]. The study of amniotic fluid-derived mensenchymal stem cells has captured the attention of researchers for several reasons. AFMSCs (amniotic fluid mesenchymal stem cells) can be collected during amniocentesis and isolated from material that would be otherwise discarded. Therefore, their use is not subject to the ethical debate that surrounds the use of embryonic stem cells. Also, as with other fetal derived stem cells, storage of AFMSCs is easy and achieved at minimal costs. AFMSC populations can be easily expanded, and have shown the capability of being stored over long periods of time with no adverse effects so amniotic fluid is a source of pluripotent and multipotent stem cells for organ regeneration.

Despite the importance of bovine species as model for *in vivo* studies, little is known about bovine MSCs (mesenchymal stem cells). They have been derived from umbilical cord blood [3], bone marrow [4,5] from attempts to provide novel insights into the *in vitro* culture and characterization of AFMSCs. However, unlike ES cells (embryonic stem cells), the AF (amniotic fluid) derived stem cells do not form teratoma when injected subcutaneously into nude mice [6]. Thus, the AF derived stem cells may be an intermediate type of cells between ES cells and adult stem cells. The aim of the present work is to isolate MSCs from AF and to characterize them in terms of morphology, specific mesenchymal or pluripotent markers, and proliferative and differentiation potential.

## 2. Results and Discussion

### 2.1. Morphological Observation of AFMSCs

**Figure 1.**
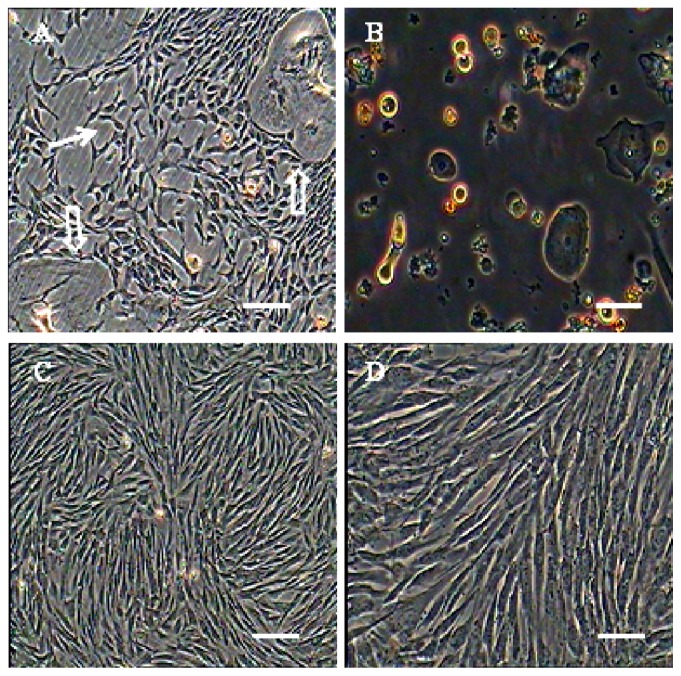
Morphology of primary cultured and subcultured AFMSCs (amniotic fluid mesenchymal stem cells). (**A**) On day 3 of culture, many cell types were mixed with the AFMSCs, the E-type cells as indicated by the hollow arrows, the AF-type cells as indicated by solid arrows; (**B**) after the cells were digested by trypsin-EDTA solution, the digestion resistant cells remained attached to the dishes; and (**C**,**D**) the passage 3–4 of AFMSCs was longer with protrusions clearly seen. Most cells had converged by this time. Scale bar = 100 μm.

**Figure 2.**
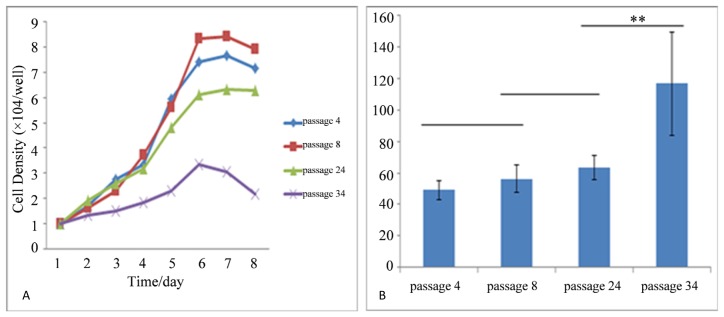
The growth curve and PDT (population double time) of AFMSCs. (**A**) Growth curves of AFMSC cultures at P4, P8, P24 and P34; (**B**) the PDT of AFMSCs was different between passages. ** *p* < 0.01.

Primary cells collected from amniotic fluid adhered to plates at 48 h later. Cells expanded rapidly shown in Figure 1A,B. The PDT (population double time) was determined to be 49, 56, 63 and 116 h for P4, P8, P24 and P34, respectively., Approximately 5–6 day later, cells were reached 70%–80% confluency, in primary cultures and, many type cells type were mixed with the AFMSCs; however, after 3–4 passages, these cells detached and were eliminated from the population and which displayed a unique vortex shape (Figure 1C,D). There were no obvious morphological differences among different passages and cellular morphology remained stable after serial passages. Cells were cultured up to passage 36 with most cells showing signs of senescence such as slow cell proliferation and vacuolization, this phenomenon is consistent with growth curve and PDT statistical analysis (Figure 2A,B). As the passage number increased, we observed more cells detaching from the culture plates.

### 2.2. Self Renewal and Proliferation Assays

The growth curve of the AFMSCs cells appeared as a typical “S” shape (Figure 2A). The PDT was calculated from the curve data and different passages statistical analysis is shown by bar chart (Figure 2B). P34 AFMSCs proliferation capability was significantly lower than P4, P8 and P24 (*p* < 0.01), but P4, P8 and P24 cells showed no obvious differences among these passages (*p* > 0.01). With increased passage numbers, AFMSCs proliferation capability declined.

**Figure 3.**
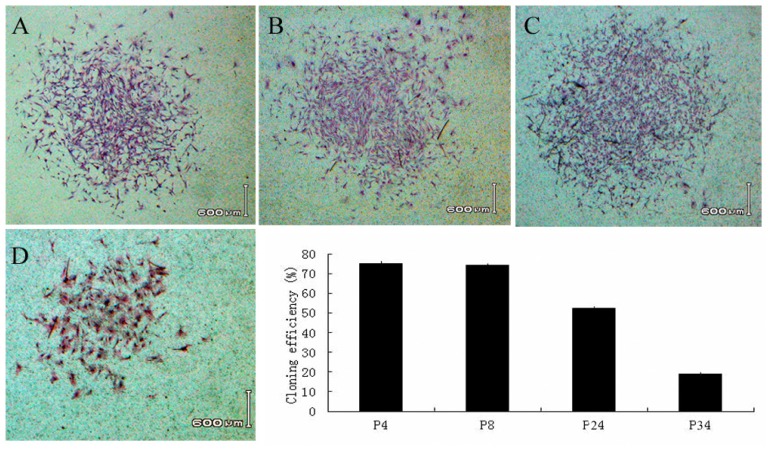
Colony forming efficiency of AFMSCs. Colonies with the morphology of AFMSCs were cultured for 2 weeks. (**A**) P4; (**B**) P8; (**C**) P24; (**D**) P34, scale bar = 600 μm. Bar chart showing the cloning rates for different passages of AFMSCs.

Colony formation was stained by Giemsa after 14 days. The colony-forming efficiency rates were 75.21% ± 0.89%, 74.19% ± 0.75%, 52.89% ± 0.78% and 18.91% ± 0.85% for passage 4, passage 8, passage 24 and passage 34 cells, respectively. These results demonstrated the self-renewal capacity of the cultured bovine AFMSCs (Figure 3).

### 2.3. Characterization of AFMSCs

**Figure 4.**
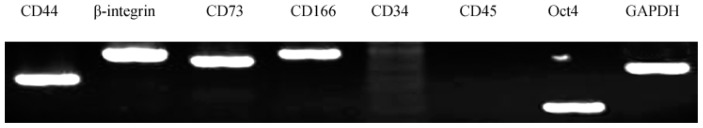
RT-PCR analysis showed that AFMSCs positively expressed CD44, β-integrin, CD73, CD106, and Oct4, but the CD34 and CD 45 were negatively expressed. GAPDH served as the internal control.

**Figure 5.**
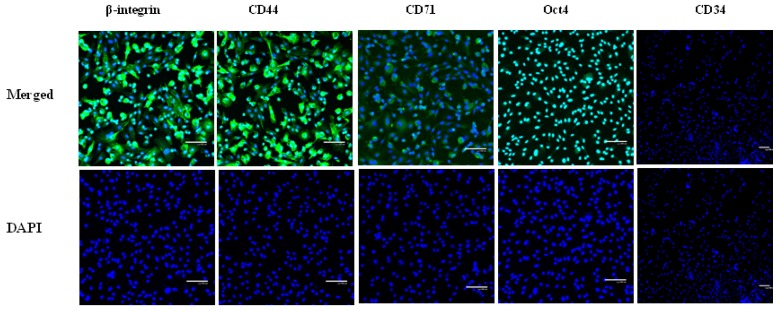
Characteristics of AFMSC surface antigens. *The first line*: Immunofluorescence showing β-integrin, CD44 CD71 and Oct 4 positive cells that were negative for CD34. *The second line*: Nuclei were stained by DAPI (4′,6-diamidino-2-phenylindole), respectively. Scale bar = 100 μm.

It has been reported that human AF derived stem cells express surface antigens including CD29, CD44, CD73 and CD166, but not CD45 and CD34 [7,8]. Based on immunocytochemistry and RT-PCR analyses, we found that the AFMSCs expressed β-integrin, CD44, CD73, CD166 and Oct-4, but not CD34 and CD45 (Figures 4 and 5). The immunofluorescence analysis of ES specific markers showed that AFMSCs expressed Oct4 demonstrating that AFMSCs has a similar feature to ES cells (Figure 4, Oct4).

### 2.4. *In Vitro* Differentiation of AFMSCs

**Figure 6.**
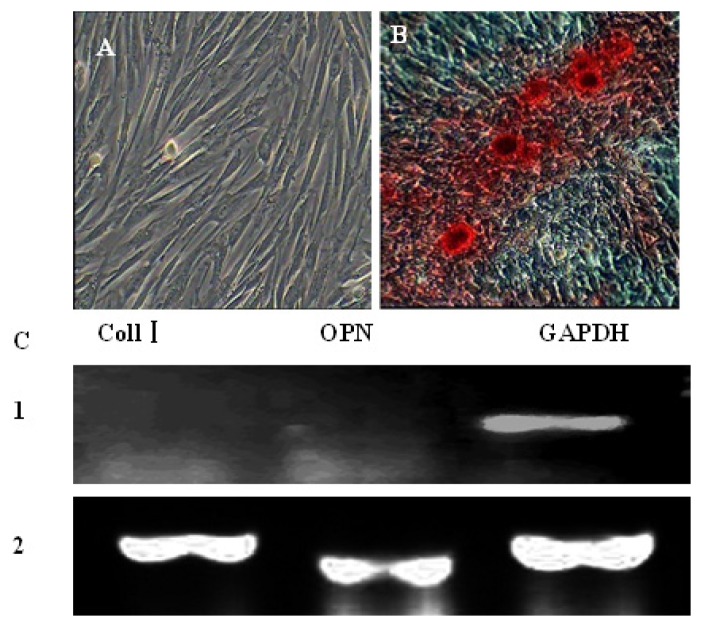
Osteogenic differentiation of AFMSCs. (**A**) Control cells; (**B**) after induction in osteogenic medium for 14 days, the cells changed from fusiform to triangular in shape and were positive for alizarin red staining. Calcified nodules increased in number and became larger during induction. After about 14 days, the nodules were observed by alizarin red staining. Cells cultured in complete medium showed no morphological changes and were negative for alizarin red staining; (**C**) after induction for 14 days, RT-PCR revealed the expression of osteoblast-specific genes Coll I and OPN in the induced group, whereas these genes were not expressed in the control group. *1*: Coll I and OPN were negative in the control group; *2*: Coll I and OPN were positive in the inducted group. GAPDH served as the internal control.

The negative control cells cultured in complete medium for the duration of the culture process were not stained by Alizarin Red staining (Figure 6A). After induction with osteogenic induction medium, AFMSCs displayed significant changes in appearance. From day 3 post-induction, the shape of some cells changed from shuttle-like to a larger, more polygonal shape. With time, the number of triangular or polygonal cells increased, growing as multiple layers, and crystalline particles became evident. At 14 days post-induction, cells were positive by Alizarin Red staining (Figure 6B). Past 14 days post-induction, the number of nodules in the culture gradually increased and expression of specific genes such as collagen type I (coll I) and osteopontin (OPN), was detected by RT-PCR (Figure 6C).

**Figure 7.**
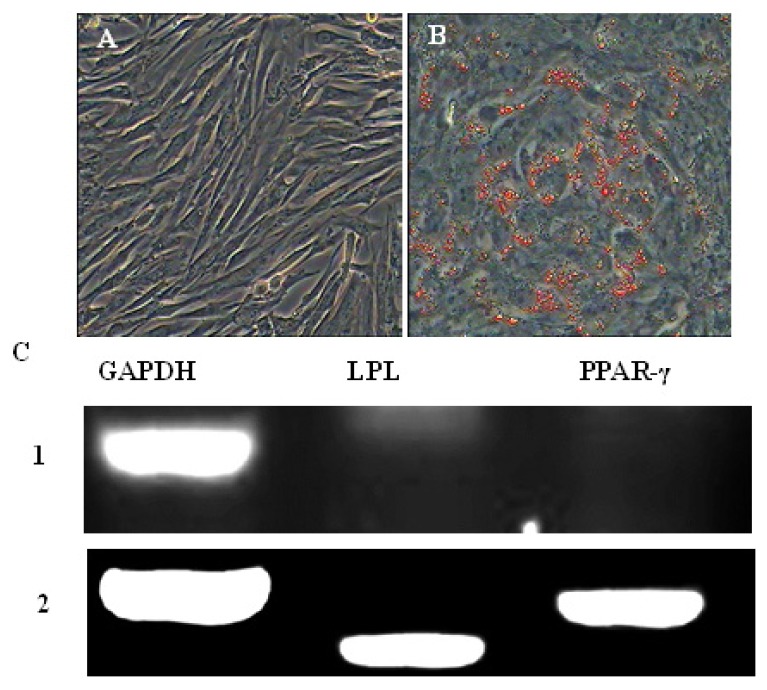
Adipogenic differentiation of AFMSCs. (**A**) As a negative control, cells cultured in complete medium showed no changes in morphology and were negative for oil red O staining; (**B**) after induction for 14 days, AFMSCs became fibroblast-like to oblate and formed many intracellular lipid droplets. Lipid droplets were stained with oil red O; (**C**) expression of adipocyte-specific genes LPL and PPAR-γ was detected by RT-PCR in the induced group after induction for 14 days. Adipocyte-specific genes were not expressed in the control group. *1*: LPL and PPAR-γ were negative in the inducted group; *2*: LPL and PPAR-γ were positive in the inducted group. GAPDH served as the internal control.

The negative control cells cultured in complete medium for the duration of the culture process were not stained by Oil Red O (Figure 7A). Adipogenic differentiation of AFMSCs was evidenced by positive Oil Red O staining. After incubation in adipogenic medium for 14 days, many lipid droplets were observed in the cells. With continued time in culture in the presence of inducers of differentiation, the number of droplets increased and small droplets aggregated to form larger ones (Figure 7B). Following induction with IBMX, insulin, indomethacin and dexamethasone, RT-PCR demonstrated the expression of the adipocyte specific genes, peroxisome proliferator-activated receptor (PPAR-γ) and lipoprotein lipase (LPL). Expression of these genes was not observed in the control group (Figure 7C).

**Figure 8.**
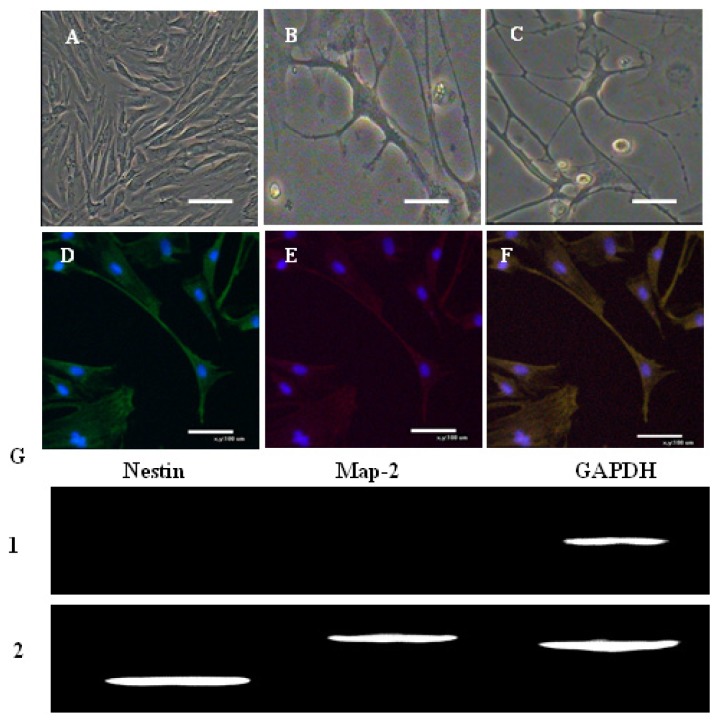
Neurogenic differentiation of AFMSCs. (**A**) Control cells; (**B**) after 1 week of induction, neural-like cells with a multipolar spindle-like shape were observed; (**C**) after 2 weeks of induction, neural-like cells were observed; (**D**–**F**) double immunofluorescence staining showed that (**D**) nestin (*green*) and (**E**) Map-2 (*red*) were positive in the induced cells; (**F**) Merged image of **D** and **E** (*yellow*). Cells were counterstained with DAPI (*blue*) (scale bar: 100 μm); (**G**) After induction for 14 days, expression of neural-specific genes nestin and Map-2 were detected by RT-PCR in the induced group, whereas these genes were not expressed in the control group. *1*: nestin and Map-2 were negative in the control group. *2*: nestin and Map-2 were positive in the inducted group. GAPDH served as the internal control. (Cy5-conjugated secondary antibody, *red*; FITC-conjugated secondary antibody, *green*).

As opposed to the fusiform morphology shown in Figure 8A, cells induced toward neural differentiation take on an elongated (Figure 8B,C), neuronal morphology and react with antibodies to nestin (green, Figure 8D) and Map-2 (red, Figure 8E). RT-PCR demonstrated positive expression of nestin and Map-2 genes in the induction group (Figure 8G, 2).

### 2.5. Discussion

In the first step, the AFMSCs were collected from the bovine amniotic fluid. At the beginning, the primary completed medium was supplemented with 20% FBS (fetal bovine serum); cells could immediately attach to the well surface and were robust and three-dimensional. Recent experiments have suggested that antigrowth factors in serum may be potential contributors to the limited proliferative capacity of cells. To reduce the concentration of inhibitory serum factors, AFMSCs were cultured in media with only 5% serum [9]. Long-term culture in 5% serum requires the addition of purified growth factors to sustain proliferation. But there are no dramatic changes in cell morphology.

A mixture of morphological aspects, limited biochemical criteria, and growth characteristics led to the classification of AFMSCs, which attach and form colonies under routine culture conditions, into three major groups: epitheloid E-type cells; amniotic fluid specific AF-type cells; and fibroblastic F-type cells (AFMSCs).

AF-type and E-type both appear at the beginning of cultivation. AF-type cells persist during the cultivation process, while E-type cells soon show a significant decrease.

For AF-type cells, 0.25% trypsin solution resistance is very poor, and after 3–4 passages, cells were purified. This result is similar to that previously reported for human amniotic fluid mesenchymal stem cells [10]. E-type cells are thought to derive from fetal skin and urine, AF-type cells from fetal membranes and trophoblasts, and F-type cells from fibrous connective tissue and dermal fibroblasts. AF-type cells produce estrogen. Also, due to the lack of hormone production, F-type cells are considered to originate from mesenchymal tissue [11].

Proliferation and self-renewal ability was investigated by cell growth curve and clonal efficiency; the PDT was calculated from the curve data and different passages statistical analysis. When the cell passage number is increased, the PDT is prolonged. AFMSCs are clonogenic by the formation of large, flattened, undifferentiated colonies containing several hundred cells. Clonal efficiency, the ability of a single cell to form a colony, is a very important defining function that demonstrates the self-renewal potential of stem cells.

AFMSCs exhibit clonal efficiency and can be differentiated *in vitro* into adipocytes, osteocytes and neurogenic cells. AFMSCs are clonogenic by the formation of large, flattened, undifferentiated colonies containing several hundred cells. Clonal efficiency, the ability of a single cell to form a colony, is a very important defining function that demonstrates the self-renewal potential of stem cells.

Dexamethasone combined with β-glycerophosphate and ascorbate was the most potent inducer of osteogenic differentiation [12]. The induction process also depended on the time of application; the most effective induction time was 14 days after the initiation of culture. In the presence of β-glycerophosphate and ascorbate, osteoblast cultures will spontaneously differentiate along a well characterized and ordered developmental pathway to form mineralized bone nodules. These nodules demonstrate the morphological and biochemical characteristics of stem cells and are useful for RT-PCR assay of osteoblast commitment and bone formation *in vitro*.

In recent years, insulin, IBMX (isobutylmethylxanthine) and indomethacin have been used to induce MSCs to differentiate into adipocytes. This study showed that combined use of IBMX and dexamethasone is able to promote adipogenic differentiation of MSCs by unknown mechanisms, probably because of the expression of PPAR-γ and LPL. Oil red O is a fat-specific reagent, and was therefore used for fat droplet detection.

The ability of MSCs to differentiate into functional neurons has been demonstrated by many *in vitro* studies [13]. Cellular differentiation by the vitamin A derivative all-trans-retinoic acid has been studied with undifferentiated pluripotent embryonic carcinoma cells [14]. *In vivo*, RA was identified as a morphogenic, teratogenic compound and, furthermore, as a signaling molecule regulating gene expression. Neurogenic cell differentiation of mesenchymal stem cells has been described by Liu *et al.* [15]. Among the many factors used to induce mesenchymal cell differentiation, all-trans-retinoic acid has an important role. RA-treated AFMSCs have been shown to give rise to a large variety of neural cells. Our results showed that AFMSCs could be induced to differentiate into neurogenic cells.

In our study, we induced AFMSCs isolated from bovine embryos to differentiate into osteoblasts, adipocytes and neurogenic cells and characterized them by the expression of genes for such cells. The results showed that different factors determined the differentiation pathway followed by the AFMSCs. The homotransplantation feature of AFMSCs together with their putative multipotency and ease of procurement, suggest that these cells are an excellent choice for many tissue engineering strategies and cell-based therapies. Further research into their differentiation mechanisms will promote the use of AFMSCs in cell therapy.

## 3. Experimental Section

### 3.1. Ethics Statement

The study was approved by the Chinese Academy of Agriculture Sciences (Beijing, China) Institutional Animal Care and Use Committee.

### 3.2. Materials

All cell culture media and supplements were from Sigma (Sigma-Aldrich, St, Louis, MO, USA), unless stated otherwise. All cell culture plates were obtained from Nest (Wuxi Nest Biotechnology Co. Ltd., Wuxi, China).

### 3.3. Isolation and Culture of the MSCs from Amniotic Fluid

Fetus samples were collected after caesarean section (4–5 month) under sterile conditions. Bovines were provided by the of the Chinese Academy of Agriculture Sciences’ farm. In this research 6 fetal bovines were used continually, which were transported to the laboratory within 4–8 h in an ice-box.

The amnion layer was punctured with a 50 mL syringe under sterile conditions for the collection of amniotic fluid that was centrifuged at 1000× *g* for 8 min. Using 100 mL sterile centrifuge tubes, the amniotic fluid was filled in the same centrifuge tube continually. Then the cell pellet was obtained and resuspended in primary cell (1–3 passage) complete medium (l-DMEM; Gibco, Carslbad, CA, USA +20% (*v*/*v*) fetal bovine serum (FBS); Gibco +5 ng/mL basic fibroblast growth factor (b FGF); Peprotech, Rocky Hill, TX, USA, +2 mM l-glutamine). Cells were plated into flasks at 1 × 10^5^ cells/mL, and incubated at 37 °C/5% CO_2_. At 48 h post-seeding, cells were washed twice with phosphate buffered saline (PBS) without calcium and magnesium to remove non-adherent cells. The cells reached 70%–80% confluency and were trypsinized (0.25% trypsin and 0.01% EDTA; *w*/*v*) to dissociate cells from the plates. Cells were subcultured onto new plates, and after 4 passages cells displayed the spindle cell type. Cells were passaged in complete medium (l-DMEM + 5% FBS + 20 ng/mL b FGF + 2 mM l-glutamine from passage 4) once all cells had dissociated to neutralize trypsin. Cells were subcultured onto new plates, and after 3–4 passages cells were purified.

### 3.4. Self-Renewal and Proliferation Assays

Cells from passages 4, 8, 24 and 34 were seeded in 60-mm plates (Wuxi Nest Biotechnology Co. Ltd.) at a density of 20 cell/cm^2^. After 14 days, the numbers of colony-forming units were counted and the cloning efficiencies were calculated as: colony forming unit number/starting cell number × 100% [16].

The cells were harvested and plated in 24-well microplates at a density of 1 × 10^4^ cells/well. The cells from three random wells were counted each day for 7 day. Growth curves were plotted according to the mean values, and the PDT calculated by the growth curve.

(1) $$\text{PDT}=(t-t_{0})\;\text{lg}2/(\text{lg}Nt-\text{lg}N_{0})$$

where *t*_0_ = starting time of culture; *t* = termination time of culture; *N*_0_ = initial cell number of culture; *Nt* = ultimate cell number of culture.

### 3.5. Characterization of AFMSCs

#### 3.5.1. Immunofluorescence

The protocol for immunocytochemistry was described previously [17]. Cells were plated at a density of 1 × 10^5^ cells/well onto 24-well plates fixed with 4% (*w*/*v*) paraformaldehyde for 1 h, and then incubated with phosphate buffered saline (PBS) containing 5% (*v*/*v*) goat serum for 30 min. Next, PBS containing 0.3% (*v*/*v*) Triton was applied and 1 μg of primary antibody in 100 μL of PBS was incubated with the cells for 1 h. FITC (fluorescein isothiocyanate)-labelled goat anti-mouse IgG antibody, FITC-labelled goat anti rat IgG antibody, FITC-labelled goat anti rabbit and Cy5-labelled donkey anti-rabbit IgG (Santa Cruz Biotechnology, Santa Cruz, CA, USA) was used at a concentration of 10 μg/mL. Primary antibodies used in this study included anti-cattle β-integrin (polyclonal IgG, 1:500; Abcam, Cambridge, MA, USA), rat anti-cattle CD44 (Monoclonal IgG, 1:1000 Abcam, Cambridge, MA, USA), rabbit anti-cattle CD71 (polyclonal IgG, 1:500; Abcam, Cambridge, MA, USA), mouse anti-cattle nestin (polyclonal IgG, 1:200; Santa Cruz Biotechnology, Dallas, TX, USA) and rabbit anti-cattle Map-2 (polyclonal IgG, 1:200; Santa Cruz Biotechnology). The cell nuclear stain DAPI was also used (Sigma-Aldrich, St. Louis, MO, USA).

Photomicropraphs were taken using a Nikon TE-2000-E confocal microscope with an attached Nikon ZE-1-C1 3.70 digital camera system (Nikon, Tokyo, Japan), and then quantified by video densitometric analysis using image software (Yokohama, Kanagawa, Japan).

#### 3.5.2. RT-PCR Assays

Total RNA was isolated using Trizol (Invitrogen, Carlsbad, CA, USA). RNA concentrations were measured by absorbance at 260 nm with a spectrophotometer and 2 μg of DNase I-treated RNA of each sample served as a template for a one-step reverse transcription-polymerase chain reaction (RT-PCR) system (Takara, Dalian, China). The RT-PCR was continued for 35 cycles after an initial denaturation at 94 °C for 10 min. Each cycle of PCR consisted of 94 °C for 30 s, annealing temperature for 30 s and 72 °C for 30 s, as well as a final extension of 10 min at 72 °C. PCR products were visualized with ethidium bromide on a 2% agarose gel. Product sizes, annealing temperatures, and primer sequences are listed in Table 1.

### 3.6. Multiple Differentiations Potential

AFMSCs at P4 were induced to differentiate into osteoblasts by culturing in l-DMEM supplemented with 5% FBS, 0.1 mM dexamethasone, 10 mM β-glycerophosphate and 50 μmol/L ascorbate for 14 day. The differentiation potential for osteogenesis was assessed by the mineralization of calcium accumulation by alizarin red staining and osteocytes specific genes were detected by RT-PCR.

Adipogenic differentiation cells were exposed to l-DMEM supplemented with 1 μmol/L dexamethasone, 5 μg/mL insulin, 0.5 mmol/L isobutylmethylxanthine (IBMX) and 60 μmol/L indomethacin for 2 weeks, with the induction medium changed every 2 day [18]. For adipogenic differentiation, intracellular lipid droplets could be observed under the microscope and confirmed by Oil Red O staining and adipocytes specific genes were detected by RT-PCR.

Neurogenic differentiation cells were seeded and divided into plates using l-DMEM medium supplemented with 5% FBS, 1 μM all-trans-retinoic acid, and 100 μM 2-mercaptoethanol [19]. After 14 days, the cells were harvested and neural specific makers [nestin and (microtubule-associated protein 2; Map-2)] were detected by immunocytochemical stain and RT-PCR.

## 4. Conclusions

AFMSCs were isolated from amniotic fluid mesenchymal stem cells obtained from 4- and 5-m-old bovine embryos. The self-renewal ability and differentiation potential of the isolated AFMSCs was evaluated *in vitro*. Our findings provide a platform for the establishment of a bovine AFMSC bank. We also propose a new method for preserving the valuable cell resources of animal models for human cell therapy.

## Figures and Tables

**Table 1.** Primer sequences used for RT-PCR.

Gene	Primers	Products (bp)
*CD44*	F: 5′-CGGAACATAGGGTTTGAGA-3′ R: 5′-GGTTGATGTCTTCTGGGTTA-3′	301
*β-integrin*	F: 5′-GAAACTTGGTGGCATCGT-3′ R: 5′-CTCAGTGAAGCCCAGAGG-3′	493
*CD73*	F: 5′-CAATGGCACGATTACCTG-3′ R: 5′-GACCTTCAACTGCTGGATA-3′	428
*CD34*	F: 5′-CCTCATCAGCTTTGCGACTT-3′ R: 5′-CCAGGAGCAAGGAGCACA-3′	314
*CD45*	F: 5′-CTACCCAACCTTCTACTCAA-3′ R: 5′-TTCACATCCAGGAGGTTC-3′	221
*CD166*	F: 5′-TATCAGGATGCTGGAAAC-3′ R: 5′-TAGCCAATAGACGACACC-3′	498
*OCT4*	F: 5′-CTCTTTGGAAAGGTGTTCAG-3′ R: 5′-GTCTCTGCCTTGCATATCTC-3′	155
*Coll I*	F: 5′-AGAAGCATGTCTGGGTAGGAG-3′ R: 5′-AGGATAGGCAGGCGAGATR-3′	358
*OPN*	F: 5′-CCAATGAAAGCCCTGAG-3′ R: 5′-TCCTCCTCTGTGGCATC-3′	310
*PPAR-γ*	F: 5′-ATCCCTGTTCCGTGCTG-3′ R: 5′-GGGATACAGGCTCCACTT-3′	356
*LPL*	F: 5′-GAACTGGATGGCGGATG-3′ R: 5′-CTGGATTCCGATACTTCGACCT-3′	256
*Nestin*	F: 5′-TGAAACACCTGTGCCAACCT-3′ R: 5′-GCTTCAGCCCACATGACTTC-3′	204
*MAP2*	F: 5′-GAGAACGGAATCAACGGAGAAC-3′ R: 5′-CCAAACAGAGTGGGAGGTGC-3′	467
*GAPDH*	F: 5′-GGCAAGTTCAACGGCACAGTCA-3′ R: 5′-TAAGTCCCTCCACGATGCCAAAG-3′	364
